# Supplementary material for: Loss of PIK3CA allows in vitro growth but not in vivo progression of KRAS mutant lung adenocarcinoma in a syngeneic orthotopic implantation model
Source: bioRxiv. 2026 Feb 4:2026.02.02.701385. Preprint. [Version 1] doi: 10.64898/2026.02.02.701385 (PMC12889443; doi:10.64898/2026.02.02.701385)
Supplement: Supplement 1 [file NIHPP2026.02.02.701385v1-supplement-1.pdf]

bioRxiv preprint doi: https://doi.org/10.64898/2026.02.02.701385; this version posted February 4, 2026. The copyright holder for this preprint (which was not certified by peer review) is the author/funder, who has granted bioRxiv a license to display the preprint in perpetuity. It is made available under aCC-BY 4.0 International license.

Supplementary data

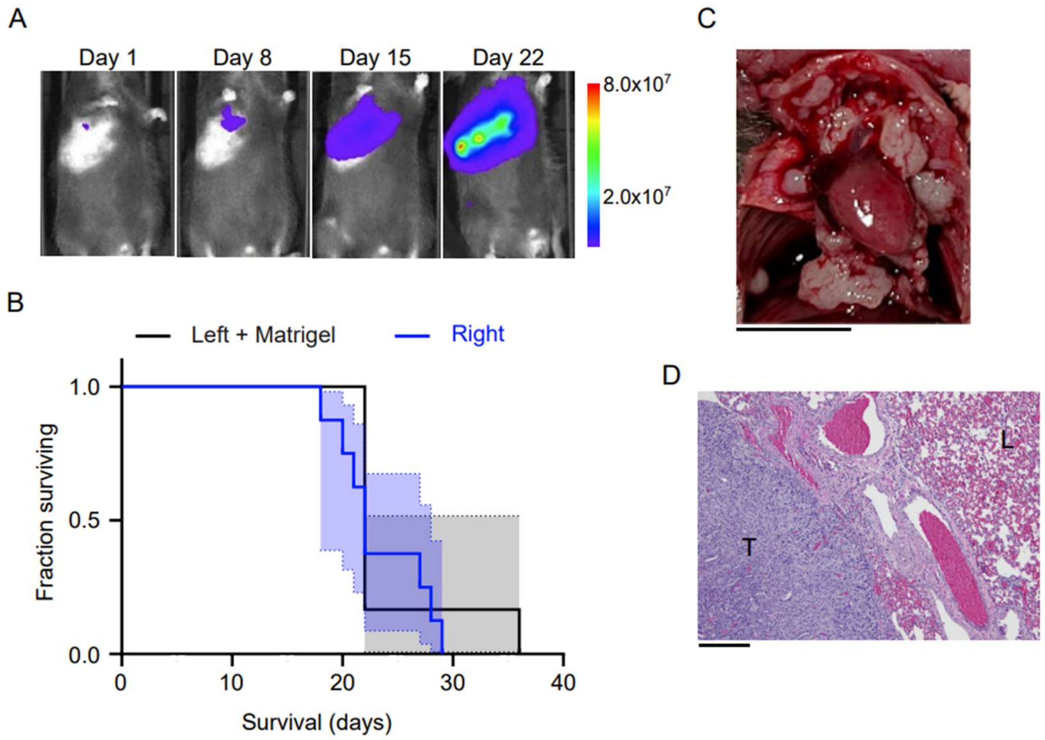

Supplemental Figure 1. In vivo LUAD model with KP cell line. $5x10^5$ KP cells were implanted into the right (n = 8) or left (n = 6) lung of mice. The cell solution injected into the left lung also contained 500 µg/mL growth factor-reduced Matrigel. (A) Tumor progression was observed with IVIS imaging at weekly intervals points post-implantation. Images from one representative mouse are shown. Scale represents luminescence intensity (arbitrary units). (B) Kaplan-Meier survival curves showing no significant difference and a median survival time of 22 days for both groups. (C) Macroscopic image of chest cavity from one mouse that died of tumor progression. Scale bar represents 10 mm. (D) H&E staining of lung section showing tumor (T) adjacent to normal lung tissue (L). Scale bar represents 200 µm.

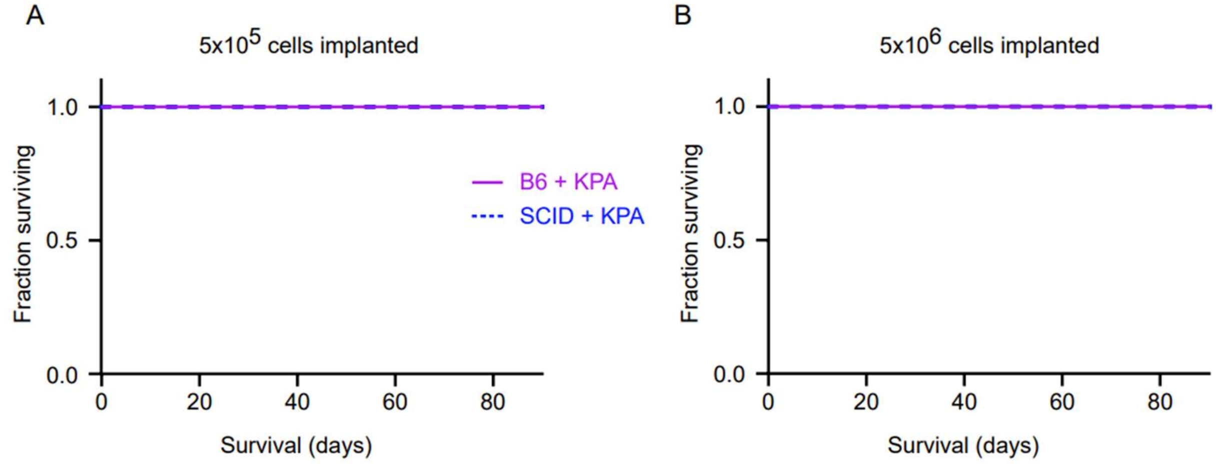

Supplemental Figure 2. Lack of KPA in vivo tumor progression is not due to adaptive immune response or seeding density. (A) Kaplan-Meier survival curves for B6 or SCID mice implanted with $5x10^5$ KPA cells or KP cells as control in lung (n = 6 per group). Median survival: B6 + KP, 38 days; all mice injected with KPA cells

bioRxiv preprint doi: https://doi.org/10.64898/2026.02.02.701385; this version posted February 4, 2026. The copyright holder for this preprint (which was not certified by peer review) is the author/funder, who has granted bioRxiv a license to display the preprint in perpetuity. It is made available under aCC-BY 4.0 International license.

were alive on day 82. $p < 0.009$, KP versus KPA-injected mice (log-rank test). (B) To test whether simply increasing the number of initial KPA cells may overcome growth limitations, B6 ($n = 6$) and SCID mice ($n = 5$) were implanted with ten-fold more ($5x10^6$) KPA cells. All B6 and SCID mice were alive on day 82 suggesting growth limitations cannot be overcome simply by increased cell numbers.
